# Sodium Tanshinone IIA Silate Inhibits High Glucose-Induced Vascular Smooth Muscle Cell Proliferation and Migration through Activation of AMP-Activated Protein Kinase

**DOI:** 10.1371/journal.pone.0094957

**Published:** 2014-04-16

**Authors:** Wen-yu Wu, Hong Yan, Xin-bo Wang, Yu-zhou Gui, Fei Gao, Xi-lan Tang, Yin-lin Qin, Mei Su, Tao Chen, Yi-ping Wang

**Affiliations:** 1 State Key Laboratory of Ophthalmology, Zhongshan Ophthalmic Center, Sun Yat-sen University, Guangzhou, China; 2 State Key Laboratory of Drug Research, Shanghai Institute of Materia Medica, Chinese Academy of Sciences, Shanghai, China; 3 Carefree Pharmaceutical Co., Ltd, Nanjing, China; Albany Medical College, United States of America

## Abstract

The proliferation of vascular smooth muscle cells may perform a crucial role in the pathogenesis of diabetic vascular disease. AMPK additionally exerts several salutary effects on vascular function and improves vascular abnormalities. The current study sought to determine whether sodium tanshinone IIA silate (STS) has an inhibitory effect on vascular smooth muscle cell (VSMC) proliferation and migration under high glucose conditions mimicking diabetes without dyslipidemia, and establish the underlying mechanism. In this study, STS promoted the phosphorylation of AMP-activated protein kinase (AMPK) at T172 in VSMCs. VSMC proliferation was enhanced under high glucose (25 mM glucose, HG) versus normal glucose conditions (5.5 mM glucose, NG), and this increase was inhibited significantly by STS treatment. We utilized western blotting analysis to evaluate the effects of STS on cell-cycle regulatory proteins and found that STS increased the expression of p53 and the Cdk inhibitor, p21, subsequent decreased the expression of cell cycle-associated protein, cyclin D1. We further observed that STS arrested cell cycle progression at the G_0_/G_1_ phase. Additionally, expression and enzymatic activity of MMP-2, translocation of NF-κB, as well as VSMC migration were suppressed in the presence of STS. Notably, Compound C (CC), a specific inhibitor of AMPK, as well as AMPK siRNA blocked STS-mediated inhibition of VSMC proliferation and migration. We further evaluated its potential for activating AMPK in aortas in animal models of type 2 diabetes and found that Oral administration of STS for 10 days resulted in activation of AMPK in aortas from *ob/ob* or *db/db* mice. In conclusion, STS inhibits high glucose-induced VSMC proliferation and migration, possibly through AMPK activation. The growth suppression effect may be attributable to activation of AMPK-p53-p21 signaling, and the inhibitory effect on migration to the AMPK/NF-κB signaling axis.

## Introduction

Diabetes mellitus is associated with increased risk of cardiovascular disease. Previous studies have reported that diabetic patients have a higher risk of developing atherosclerotic cardiovascular disease and higher rate of restenosis after percutaneous coronary intervention (PCI), compared to normal subjects [1]. Diabetes itself is an important risk factor for accelerated development of atherosclerosis. High blood glucose is a key factor that induces cardiovascular complications, including hypertension and atherosclerosis [2]. VSMC proliferation, extracellular matrix production, and decreased programmed cell death are three known mechanisms of restenosis. In response to FBS, proliferation and DNA synthesis of VSMCs have been shown to be significantly enhanced under high glucose (25 mM glucose, HG) versus normal glucose conditions (5.5 mM glucose, NG) [3]. Additionally, Zhu and colleagues demonstrated that glucose induced VSMC proliferation and migration in a dose-dependent manner [4], similarly, we applied the same model to mimic a hyperglycemic (diabetic-like) state.

Adenosine 5′-monophosphate-activated protein kinase (AMPK), a physiological sensor of cellular energy status, plays a crucial role in regulating lipid homeostasis and whole body glucose, and thus presents a potential target for treating obesity and diabetes [5]. AMPK additionally exerts several salutary effects on vascular function and improves vascular abnormalities. For example, AMPK activation leads to improved endothelial function [6], attenuation of myocardial ischemia injury in mice [7], and suppression of VSMC proliferation and migration as well as neointimal formation in a balloon injury rat model [8], [9]. Additionally, Ning et al. [10] demonstrated that regulation of AMPK inhibited VSMC proliferation and migration that occur in response to chronic hyperglycemia. HG impairs AMPK activation in VSMCs, and exposure to 25 mM glucose led to significant decrease in phosphorylation of AMPK at T172.

Danshen is an important source of a large number of active natural compounds which are mainly classified as aqueous and lipid soluble (diterpenes) fractios [11]. Tanhinone IIA is the most active diterpenoid quinine pigment in danshen. Sodium Tanshinone IIA silate (STS) is a water-soluble derivative of tanshinone IIA. STS accepts electrons from complex I to convert to a semiquinone form, which reduces the oxygen molecule [12]. The beneficial effects of tanshinone IIA on diabetes have been demonstrated in previous studies. Notably, tanshinone IIA reduces adipose mass and body weight, improves glucose tolerance, and lowers the low-density to high-density lipoprotein ratio without altering food intake in a high-fat diet induced obese animal model [13]. Similar results have been reported with *db/db* mice, whereby tanshinoe IIA reduced blood glucose levels and body weights [14]. Moreover, pretreatment with tanshinone IIA reduced infarct size and improved cardiac dysfunction after ischemia/reperfusion injury in diabetic rats [15]. Tanshinone IIA has additionally been shown to abolish VSMC proliferation and reduce intimal hyperplasia [16]. However, no studies to date have documented the effect of STS under high glucose conditions mimicking diabetes.

Owing to the higher incidence of atherosclerosis in diabetes, the effects of STS on diabetes without dyslipidemia have yet to be established. Tanshinone IIA has been shown to enhance AMPK phosphorylation in endothelial cells [7], liver protein isolated from HF diet induced obese mice [13], L6 myotubes and skeletal muscle tissue of *db/db* mice [14]. Here, we determined the effects of STS on VSMC proliferation and migration under high glucose conditions mimicking diabetes, and further investigated the underlying molecular mechanism, focusing on signaling pathway for AMPK activation.

## Materials and Methods

DMEM cell culture medium was obtained from Gibco Inc. (US). Antibodies for matrix metalloproteinase-2 (MMP-2), CyclinD1 and p21 were purchased from Abcam Inc. (UK), and those for AMPK, phosphor-AMPK, p53, and β-actin were supplied by Cell Signaling Technology Inc. (US). All reagents were purchased from Sigma, unless otherwise specified.

### Ethics statement

All procedures performed on animals were conducted in strict accordance with the Guide for the Care and Use of Laboratory Animals (Institute of Laboratory Animal Resources, Commission on Life Sciences, National Research Council), and experimental protocols were approved and supervised by Institutional Animal Care and Use Committee (IACUC) of Shanghai Institute of Materia Medica, Chinese Academy of Sciences. All efforts were made to improve animal welfare and minimize suffering.

### Cell culture and treatment

Primary vascular smooth muscle cells were isolated from the thoracic aortas of Sprague-Dawley rats, and characterized morphologically and immunohistochemically, as described previously [17]. Growth-arrested VSMCs were incubated with or without STS in DMEM containing normal (5.5 mM,NG) or high glucose (25 mM,HG). High glucose DMEM represents normal medium added with 19.5 mM glucose (1000 mM, dissolved in PBS and sterilized via filtration) to generate a final concentration of 25 mM glucose. VSMCs were treated with HG plus STS for 48 h before cell proliferation and migration assays or 24 h before Western blot experiments, unless otherwise specified.

### Cell proliferation assay

The 3-(4,5-dimethylthiazol-2-yl)-2,5-diphenyltetrazolium bromide (MTT) assay is based on cleavage of MTT by mitochondrial dehydrogenases, reflecting cell viability. After VSMCs were treated with STS for 48 h, 0.5 mg/mL MTT was added for 4 h at 37°C. MTT-containing medium was removed, dimethyl sulfoxide added, and absorbance examined at 570 nm.

### Flow cytometry analysis of cell cycle

Cells were seeded in 60 mm dishes and allowed to grow to subconfluence, followed by growth arrest via serum deprivation for 12 h. After relief from the quienscent state, cell cycle distribution was analyzed using flow cytometry. At high glucose levels, cells were treated with various concentrations of STS at 37°C for 48 h. Next, VSMCs were collected and washed twice with cold PBS. Cell pellets were fixed in 70% ethanol and stored at 4°C overnight. Next, cells were stained for DNA content with 0.5 ml cold staining solution containing 50 µg/ml propidum iodide (PI) and 20 µg/ml RNase A for 30 min at 37°C, and aliquots (1×10^4^ cells) analyzed using flow cytometry. The rates of G_0_/G_1_, S and G_2_/M phases were determined using the ModiFit LT program.

### Cell transfection

The cyclin D1 promoter construct (pGL3-cyclinD1) was a gift from Professor De-Pei Liu (Chinese Academy of Medical Sciences and Peking Union Medical College). VSMCs were transiently transfected with pGL3-cyclinD1 or *Ampkα1* and *Ampkα2* small interfering RNAs (siRNA, 50 nM) using Lipofectamine 2000 reagent (Invitrogen). At 24 h after transfection, cells were treated with HG with or without STS for 24 or 48 h, harvested, and luciferase activities measured with a luminometer (PerkinElmer) using the Luciferase Reporter Gene Assay Kit of Beyotime (China). AMPK protein levels were measured using Western blot analysis, the cell proliferation and migration with the MTT and transwell assay, respectively. Each experiment was performed in triplicate.

### Gelatin zymography

Gelatinase activity in conditioned medium collected from cell cultures was measured with zymography, as described previously [17]. Conditioned medium were electrophoresed on a polyacrylamide gel containing 0.1% (w/v) gelatin. After electrophoresis, the gel was washed at room temperature for 30 min with 2.5% Triton X-100, and placed overnight in incubation buffer [50 mM Tris-HCl (pH 7.4), 200 mM NaCl, and 10 mM CaCl_2_]. The gel was stained with 0.2% Coomassie brilliant blue R-250 in a mixture of methanol:acetic acid:water (2∶1∶7, v/v/v) for 2 h, and destained in the same solution without dye. Clear zones against a blue background indicated the presence of gelatinolytic activity.

### Isolation of cytoplasmic and nuclear proteins

Cytoplasmic and nuclear proteins were extracted using NE-PER Nuclear and Cytoplasmic Extraction Reagents from Thermo Scientific (US), according to the manufacturer's instructions.

### Western blot analysis

After various treatments, cells were washed twice with PBS and lysed in lysis buffer (RIPA) containing protease and phosphatase inhibitor cocktails (MERK). Whole-cell lysates were prepared by scraping cells into RIPA buffer and clarified by centrifugation at 12,000 g for 15 min at 4°C. Equal amounts of protein from each sample were electrophoresed on a 10% polyacrylamide SDS gel, followed by transfer to a polyvinylidene difluoride membrane (BioRad), and immunoblotting performed. Membranes were blocked with 5% non-fat milk in TBST buffer (50 mM Tris, pH 7.5, 250 mM NaCl, 0.1% Tween 20) and probed with the indicated antibodies overnight at 4°C. After five washes in TBST, the membranes were exposed to the appropriate secondary antibodies for 2 h at room temperature. Immunoreactive bands were visualized using chemiluminescent detection reagents, according to the manufacturer's instructions.

### VSMC migration assay

VSMC migration was examined using a transwell cell culture chamber with gelatin-coated polycarbonate membrane, as described previously, with slight modification [18]. Different stimulants were added to quiescent VSMCs for 48 h, which were subsequently detached via trypsinization and suspended in DMEM-0.5% FBS to a concentration of 4×10^5^ cells/mL. DMEM (0.6 mL) was added to the lower compartment. A 0.1 mL cell suspension (final concentration, 4×10^4^ cells/well) was added to the upper chamber precoated with 0.1% gelatin, and cells incubated at 37°C (95%air-5%CO2). After 6 h, non-migratory cells were removed from the upper surface of the membrane by scraping with cotton swabs. The membrane was fixed with 4% paraformaldehyde and stained with 2 µg/mL Hochest33342. Images of fluorescent-labeled invasive cells were additionally obtained under a fluorescence microscope. Migrating cells were counted at 200× magnification in nine randomly selected microscope fields per filter invasion assay.

### Oral administration of STS

Male *ob/ob* (IACUC:2011-12-LY-13) or *db/db* (IACUC:2011-12-LY-12) mice were housed in standard cages (6 mice per cage containing toys) in a temperature (23±2°C), humidity (40–70%) controlled and Specific Pathogen-Free room with 12 h dark/12 h light cycles. Eight week-old *ob/ob* or *db/db* were randomly divided into two groups (n = 6 per group). One group received STS (30 mg/kg, p.o.) once daily, while the other group received vehicle. After 10 days of treatment, mice were anesthetized by pentobarbital and sacrificed. Following isolation of aortas, Western blot analysis performed.

### Statistical analysis

Results are expressed as mean values ± SD. Statistical analysis was performed with one-way analysis of variance (ANOVA) or Student's *t*-test. A *p*-value of <0.05 was considered significant.

## Results

### STS stimulates AMPK phosphorylation in VSMCs

**Figure 1:**
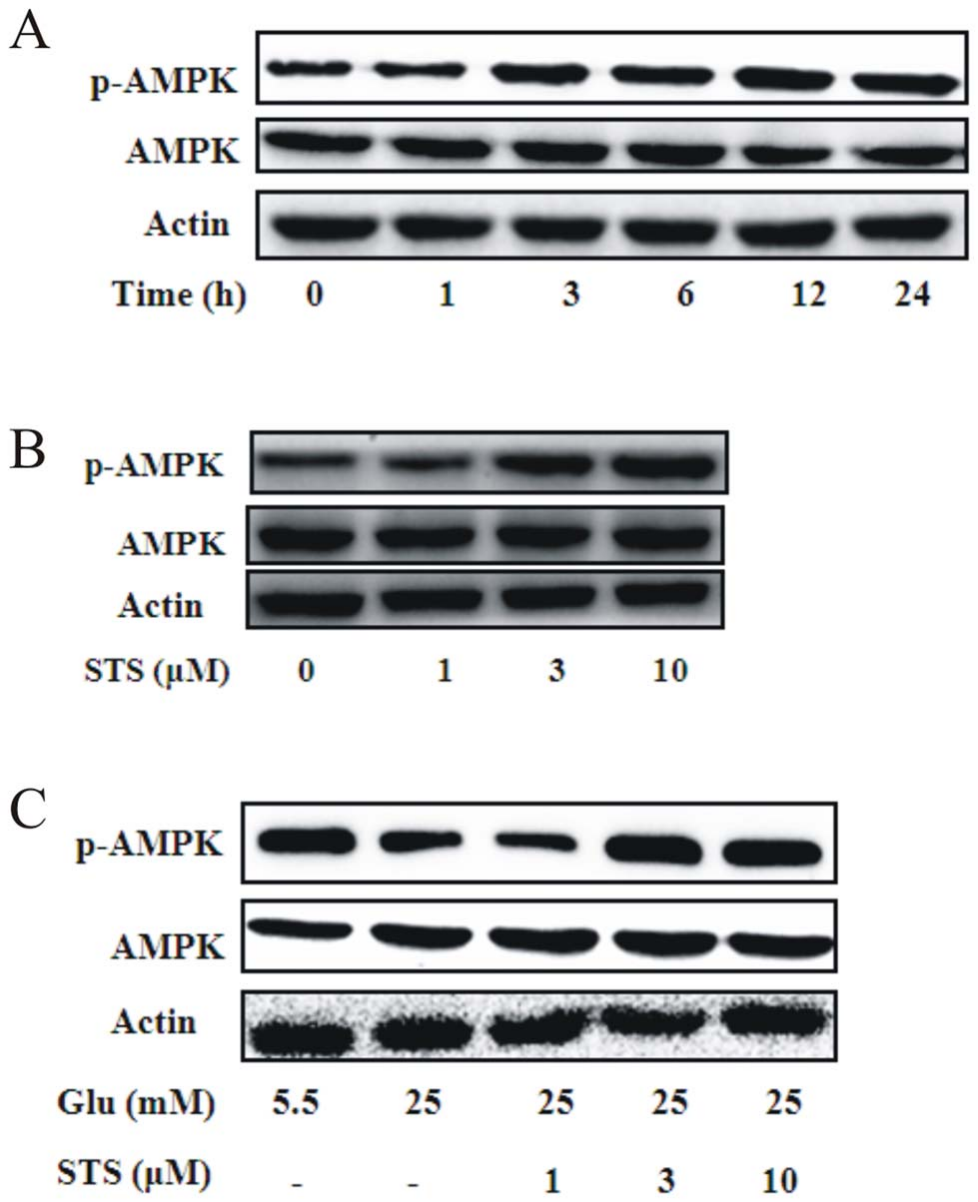
Effect of STS on phosphorylation of AMPK in VSMCs. (A) VSMCs were incubated with 10 µM STS for the indicated times in 5.5 mM glucose (NG). Protein expression of p-AMPK was determined by Western blot analysis. Results are representative of three independent experiments. (B) VSMCs were treated with the indicated concentration of STS for 24 h. Protein expression of p-AMPK was determined using Western blot analysis. Results are representative of three independent experiments. (C) VSMCs were treated with high concentrations of glucose (25 mM, HG) for 24 h in the presence of indicated concentrations of STS. Protein expression of p-AMPK was determined using Western blot analysis. Results are representative of three independent experiments.

To assess the activation effects of STS on AMPK, VSMCs were incubated with a fixed dose of STS (10 µM) for the indicated periods or with different concentrations of STS for 24 h. Western blot analyses revealed significant activation of AMPK in VSMCs by STS in a time- (Figure 1A) and dose-dependent manner (Figure 1B). Moreover, high glucose (25 mM) suppressed AMPK activation in VSMCs, consistent with previous results [10], while STS treatment enhanced AMPK phosphorylation at T172 under high glucose conditions in a dose-dependent manner (Figure 1C). Our findings clearly indicate that STS stimulates AMPK and reverses impaired AMPK activation induced by HG conditions mimicking diabetes in VSMCs.

### STS inhibits high glucose-induced VSMC proliferation

**Figure 2:**
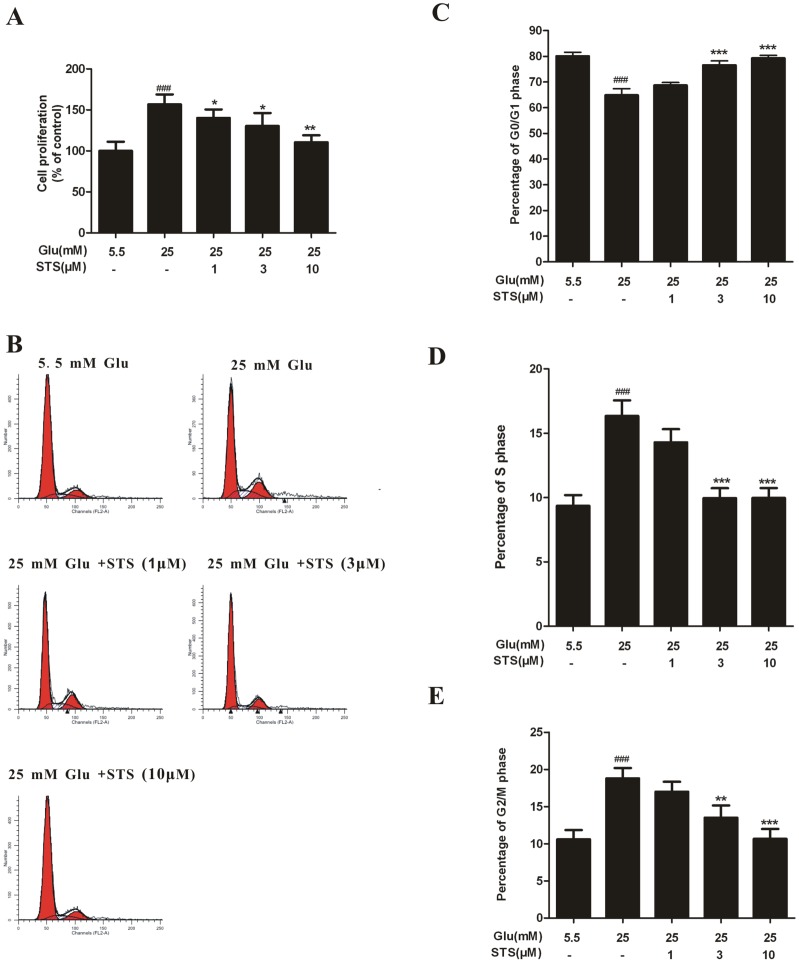
Effects of STS on cell proliferation and cell cycle distribution. (A) STS inhibits HG-induced VSMC proliferation. VSMCs were treated with different concentration of STS for 2 h before exposed to HG for 48 h. Cell proliferation was evaluated with the MTT assay. Relative proliferation activities were expressed using untreated control cells as a standard. Values are presented as mean values ± SD. ^###^ *p*<0.001 *vs* control. ^*^ *p*<0.05, ^**^ *p*<0.01 *vs* HG group. (B) VSMC counts in the G_0_/G_1_, S, and G_2_/M phases are shown for normal, high glucose, and different doses of STS. Quantitative assessment of the percentage of VSMCs at G_0_/G_1_, S, and G_2_/M phases is shown (C–E). G_0_/G_1_ phase is represented by the first peak, S phase in diagonal and and G_2_/M phase by the second peak. Cell counts were increased in the G_0_/G_1_ phase and deceased in S and G_2_/M phases, compared with the high glucose group, indicating that STS induces cell cycle arrest at the G_0_/G_1_ phase in a dose-dependent manner. Values are presented as means ± SD. ^###^ *p*<0.01 *vs* control. ^**^ *p*<0.01, ^***^ *p*<0.01 *vs* HG group.

Abnormal VSMC growth is a key mechanism underlying many vasculopathies, including atherosclerosis, vessel hypertrophy, and restenosis following injury or clinical intervention [19], [20]. We examined the effect of STS on HG-induced VSMC proliferation using the MTT assay. Exposure to HG for 48 h stimulated the proliferation of VSMCs, compared to the effects of 5.5 mM glucose. STS suppressed VSMC proliferation induced by HG in a concentration-dependent manner (figure 2A). To further investigate the relationship between STS and the cell cycle under HG conditions, we analyzed the effect of STS on the cell cycle profile in rat VSMCs using flow cytometry. Typical flow cytometry DNA histogram analysis revealed that STS induced accumulation of cells at G_0_/G_1_ phase in dose-dependent manner, while cell numbers at the S and G_2_/M phases were significantly decreased (figure 2B–E). These results suggest that STS inhibits high glucose-induced VSMC proliferation by inducing cell cycle arrest at the G_0_/G_1_ phase.

### STS efficiently abrogates high glucose-induced cyclin D1 expression and promoter activity

**Figure 3:**
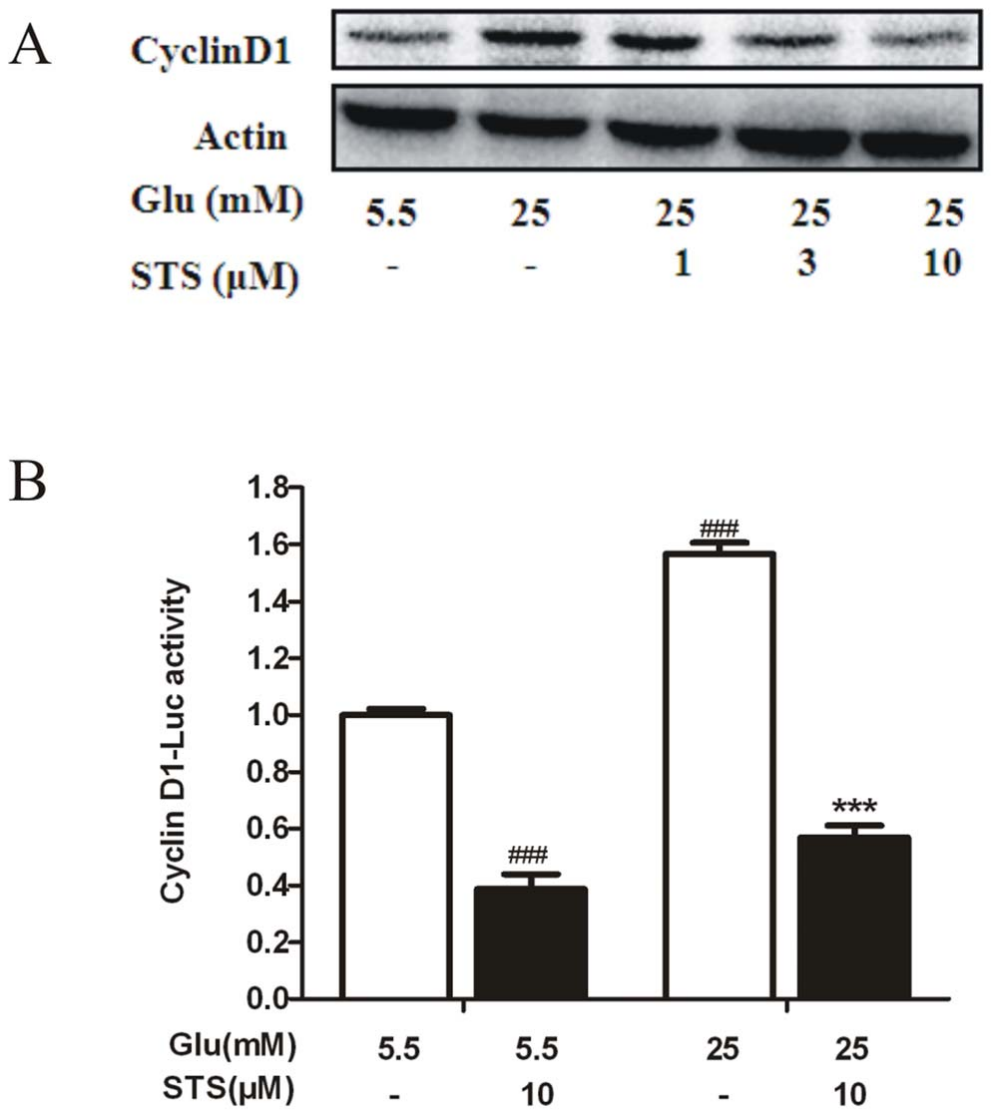
Effect of STS on HG-induced cyclin D1 expression. (A) VSMCs were treated with HG for 24 h in the presence of the indicated concentration of STS. Cyclin D1 expression was analyzed by Western blot, with β-actin as a loading control. Results are representative of three independent experiments. (B) STS decreases HG-induced cyclin D1 promoter activity. Cells were transiently transfected with pGL3-cyclin D1, and cultured with 25 mM glucose in the presence or absence of STS (10 µM) for 24 h. Luciferase activity was determined from cell lysates, as described in “Materials and methods.” Values are presented as means ± SD. ^###^ *p*<0.001 *vs* control. ^***^ *p*<0.001 *vs* HG group.

Cyclin D1 regulates the G1/S check point and has a crucial role in the development of vascular proliferation diseases [21]. We examined the expression of cell-cycle regulatory molecules using western blot. The expression of G1-associated factor cyclinD1 was up-regulated in high glucose status, while STS abolished this action (Figure 3A). In addition, we used a plasmid containing a luciferase reporter gene to examine high glucose-mediated cyclin D1 promoter activation in VSMCs. The result showed that high glucose was able to activate the pGL3-cyclin D1 in VSMCs, and STS decrease the luciferase activity. Moreover, and decrease in transcriptional activity from the cyclin D1 promoter in STS treated VSMCs was also found in basal levels (Figure 3B).

### STS induces p53 and p21 expression

**Figure 4:**
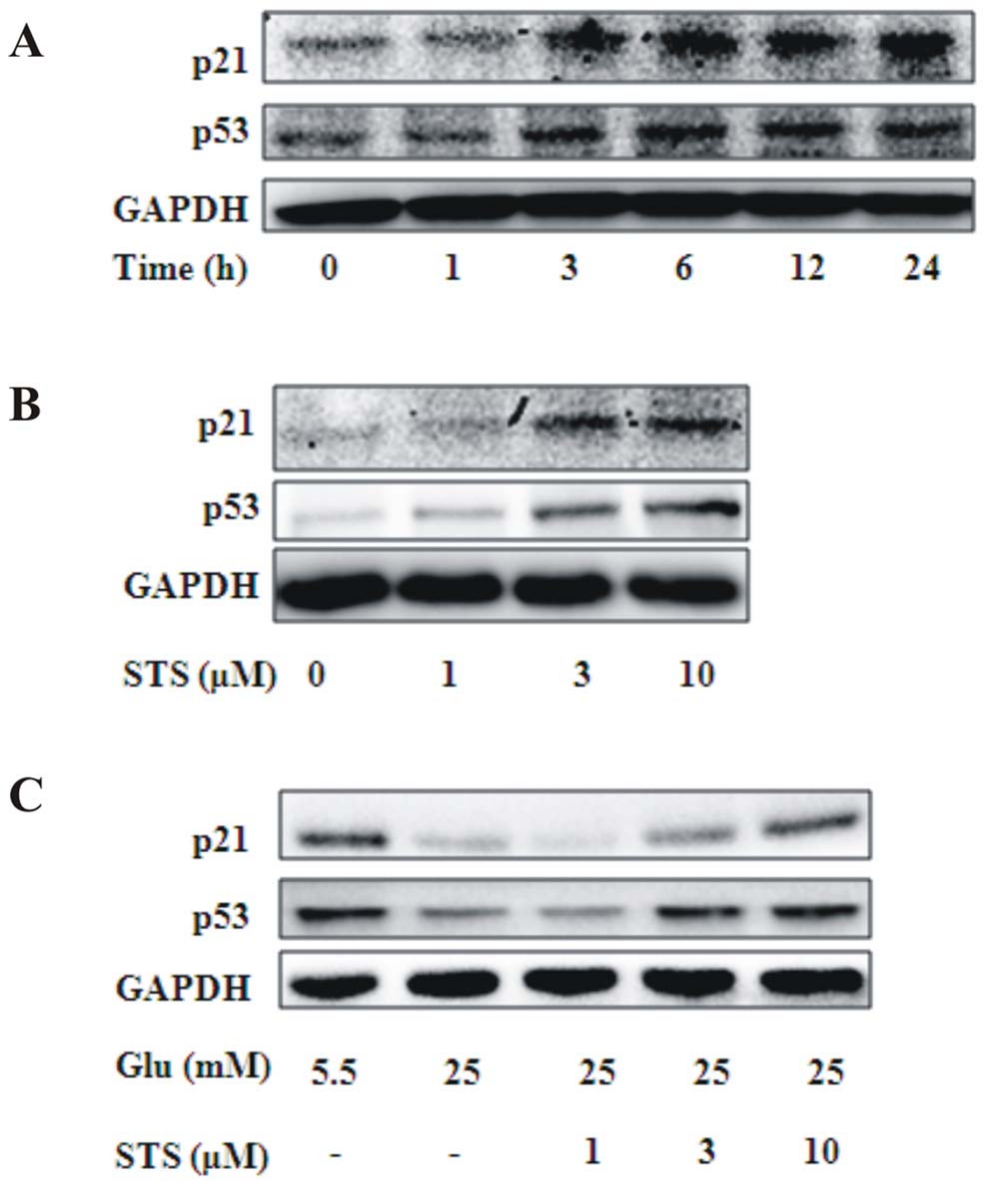
Effects of STS on cyclin-dependent kinase inhibitory proteins. (A) VSMCs were treated with STS (10 µM) for the indicated time-periods. Protein levels of p53, p21, and β-actin were determined using Western blot. (B) VSMCs were treated with different concentrations of STS for 24 h, and levels of p53, p21, and β-actin were deterimined. (C) VSMCs were treated with HG for 24 h in the presence of indicated concentration of STS. Expression levels of p53, p21, and β-actin were analyzed using Western blot. Results are representative of three independent experiments.

Previous studies have demonstrated that the mechanism of cell cycle arrest by AMPK activation involves accumulation of p53, which, in turn, upregulates p21 via a transcriptional mechanism [19]. p53, a key regulator of the cell cycle, and p21, a downstream target of p53, has been shown to downregulate cyclin D1, which is involved in cell cycle arrest [9], [22]. Hence, we focused on whether the inhibitory activities of STS on VSMC proliferation and cyclinD1 expression were regulated by the p53/p21 pathway. Western blot experiment revealed that STS enhanced p53 and p21 expression in a time- and dose-dependent manner (Figure 4A, 4B). Expression levels of p53 and p21 were low under conditions of high glucose stimulation, and promoted by STS in a dose-dependent manner (Figure 4C).

### STS inhibits high glucose-induced migration of VSMCs, attenuates enzymatic activity and expression of MMP-2, and suppresses NF-κB translocation

**Figure 5:**
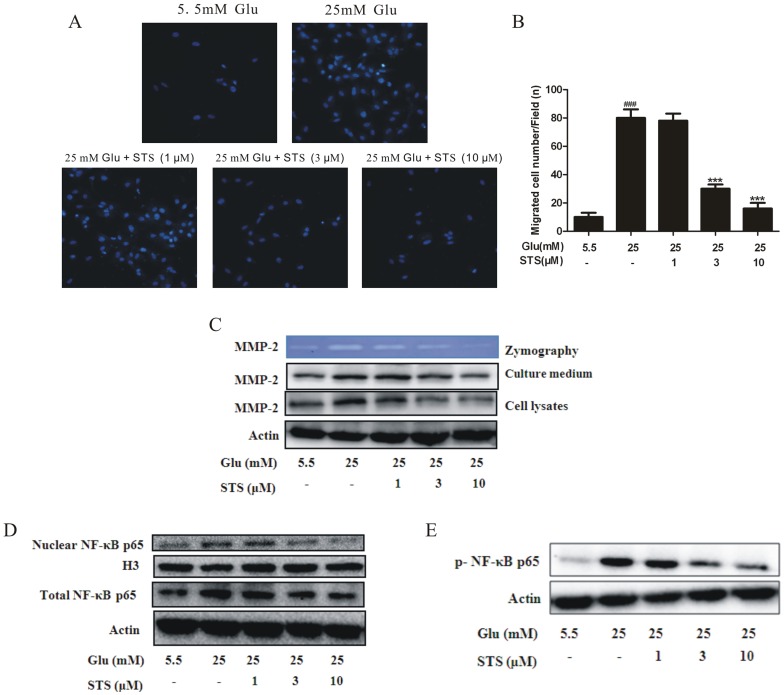
Effects of STS on HG-induced VSMC migration as well as MMP-2 and nuclear NF-κB. (A) Cells were treated as described in the Materials and methods section. Cells migrating to the lower surface of the membranes were fixed with 4% paraformaldehyde and stained with 2 µg/mL Hochest33342. (B) The diagram depicts cells counted on the bottom side of the transwell chamber membrane. Values are presented as means ± SD. ^###^ *p*<0.001 *vs* control. ^***^ *p*<0.001 *vs* HG group. (C) Quiescent cells were stimulated with HG in the presence or absence of STS for 24 h, and culture supernatants analyzed zymographically for MMP activity. Similarly, immunoblot analysis was performed with antibodies specific for MMP-2. Results are representative of three independent experiments. (D) Quiescent cells were stimulated with HG in the presence or absence of STS for 24 h, and cell lysates and nuclear extracts prepared. The levels of nuclear and total p65 were determined using Western blot. Results are representative of three independent experiments. (E) Cytoplasmic extracts were subjected to Western blot with anti-p-NF-κB p65 and β-actin antibodies. Results are representative of three independent experiments.

Activated AMPK exerts inhibitory effects on VSMC proliferation as well as migration [23], [24]. The transwell cell culture chamber was used to evaluate the migration of VSMCs under high glucose concentration. The number of cells on the bottom side of the transwell chamber was markedly increased under high glucose conditions. STS decreased the number of cells migrating into the lower transwell chamber (Figure 5A). Quantitative analysis of cell motility further showed that STS suppressed motility in a dose-dependent manner (Figure 5B). The extracellular matrix (ECM) provides a substrate for cellular binding as well as a material on which to exert forces necessary for movement. Growth factors and prosynthetic cytokines promote the generation and secretion of MMPs that degrade ECM and regulate cell motility, and the gelatinases, MMP-2 and MMP-9, are integral to VSMC migration [25]. Expression and activity levels of MMP2, but not MMP-9, were upregulated in response to high glucose (data not show). Under HG conditions, STS suppressed both expression and activity of MMP2 in a dose-dependent manner (Figure 5C). NF-κB plays a central role in inflammation, atherosclerosis and angiogenesis, and is thought to regulate angiogenesis via matrix metalloproteinase (MMP) induction. VSMC migration under HG conditions is dependent on increased MMP-2 activity [26], [27]. In our experiments, STS inhibited the expression and translocation of NF-κB induced by HG in a dose-dependent manner (Figure 5D). Activation of NF-κB is controlled by an inhibitory subunit, inhibitor of κB (I-κB), which retains NF-κB in the cytoplasm by physically masking its nuclear translocation signal. Dissociation of the cytoplasmic complex of NF-κB and IκB leads to release of active NF-κB, which translocates into the nucleus to activate target enhancers [28]. Active NF-κB has been detected in the cytoplasm under a number of conditions [28], [29]. These findings, along with the reversibility of NF-κB activation *in vivo* [29], suggest that the protein translocates freely in and out of the nucleus. In order to investigate whether STS indeed affected the translocation of NF-κB, we detected the active NF-κB in the cytoplasmic contents and found that cytoplasmic NF-κB release after HG treatment, which facilitated its translocation into nucleus, was reversed by STS in a dose-dependent manner (Figure 5E).

### STS inhibits high glucose-induced VSMC proliferation and migration via AMPK activation

**Figure 6:**
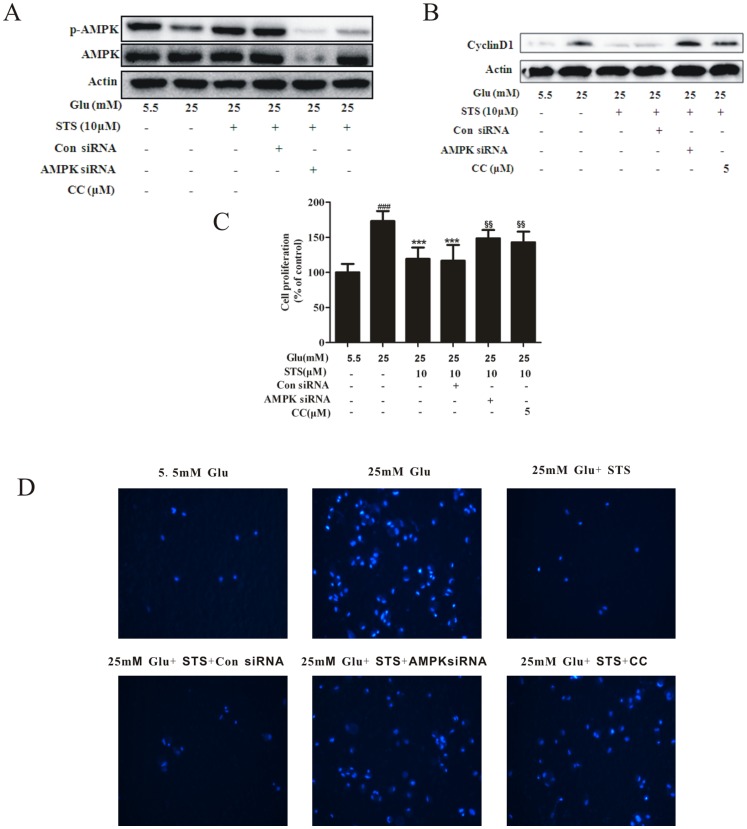
STS inhibits VSMC proliferation and migration through AMPK activation. VSMCs were transfected with AMPK siRNA for 24-treated with compound C (CC, 5 µM) or AMPK siRNA or con siRNA with STS (10 µM), and (A and B) stimulated with HG for 24 h, protein expression of p-AMPK and cyclin D1 were determined with Western blot analysis, results are representative of three independent experiments; (C) after stimulation with HG for 48 h, cell proliferation was determined with the MTT assay, n = 6, Values are presented as means ± SD, ^###^ *p*<0.001 *vs* control. ^***^ *p*<0.001 *vs* 25 mM glucose group. ^$$^ *p*<0.01 *vs* HG + STS group; (D) stimulated with HG for 48 h. Migration assays were performed, as described in the Materials and methods section. Results are representative of three independent experiments.

To further demonstrate the interactions between STS and AMPK, we examined the effects of co-administration of AMPK inhibitors and STS. Under HG conditions, STS-induced AMPK phosphorylation in VSMCs was signigicantly inhibited upon pretreatment with compound C (CC, 5 µM) or AMPK siRNA (Figure 6A). As we know, AMPK induces cell cycle arrest in a p53-Dependent manner [30], and we have found STS induced p53 and p21 expression, consistent with the trend of AMPK activation (Figure 1 and Figure 4). And cyclin D1 regulates the G1/S check point and has a crucial role in the development of vascular proliferation diseases [21]. We further investigated the effect of STS on cyclin D1 during AMPK inhibition and found that the effect of STS on cyclin D1 was restored by compound C or AMPK siRNA (Figure 6B). To further demonstrate the interaction of STS and AMPK activation, we examined the effects of AMPK inhibitors on VSMC proliferation and migration, and found that STS-mediated inhibition of HG-induced VSMC proliferation and migration was reversed upon pretreatment with CC (Figure 6C and D). These results clearly indicate that STS activates AMPK, which contributes to inhibition of VSMC proliferation and migration.

### STS induces AMPK activation *in vivo*

**Figure 7:**
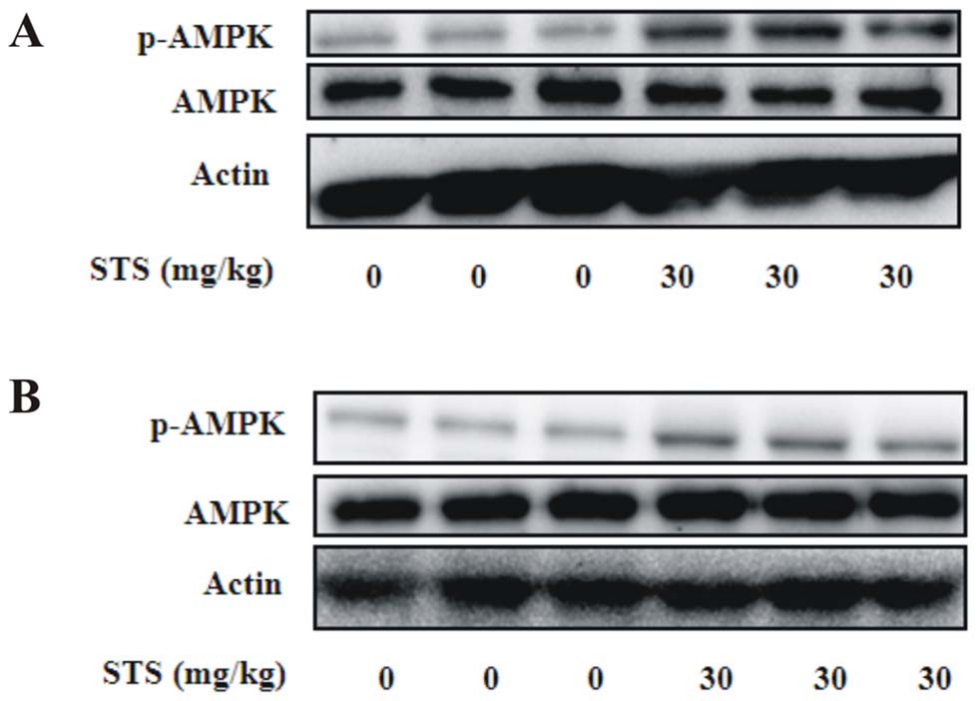
Effect of oral administration of STS on phosphorylation of AMPK in aortic tissue. Samples were prepared from aortas of male (A) *ob/ob* or (B) *db/db* mice. STS (30 mg/kg) was administered orally for 10 days. n = 6 for each group.

Next, we examined whether STS activated AMPK in the aorta using previously published oral doses of STS administered to diet-induced obese mice [13] and atherosclerotic experimental setting [31] as references. We selected a dose with reported potential in improving metabolic disorders and inhibiting neointima formation. Oral administration of 30 mg/kg STS for 10 days led to significantly increased phosphorylation of AMPK with no changes in total AMPK protein levels in *ob/ob* (Figure 7A) and *db/db* mice (Figure 7B). These findings indicate that STS activates AMPK in aortic tissue *in vivo*, similar to that observed in cultured VSMCs.

## Discussion

Our study is particularly important in light of the clinical findings that obesity and diabetes are important predictors of cardiovascular morbidity and mortality [32]. Tanshinone IIA, extracted from Danshen, a popular medicinal herb used in traditional Chinese medicine, exhibits a variety of cardiovascular activities [11]. Data from our experiments showed that STS inhibited high glucose-induced VSMC proliferation and migration. We further investigated the mechanisms underlying STS-mediated inhibition of VSMC proliferation and migration in high glucose conditions mimicking diabetes without dyslipidemia, which is distinct from previously studied models. Excessive accumulation of VSMCs is critical in the pathogenesis and progression of macrovascular diseases, and high glucose accelerates the process by promoting cellular proliferation [3] and migration [4]. Earlier studies have reported that high glucose upregulates connective tissue growth factor expression [33] and induces NF-κB activation [34] in VSMCs, leading to cell dysfunction. The ability of STS to prevent acceleration of atherosclerosis in diabetic condition may thus be partly explained by its capacity to inhibit VSMC proliferation and migration.

AMPK activation has been shown to induce inhibition of neointimal formation [8], [22] and remodeling [23] after wire-induced injury. Earlier *in vivo* studies demonstrated that tanshinone IIA improves glucose tolerance, insulin sensitivity and glucose metabolic disorders via increasing AMPK activity [13], [14]. However, to our knowledge, no studies have clarified the effects of STS on AMPK in VSMCs to date. Accordingly, in the present study, we focused on the effects of STS on AMPK and subsequent consequences in VSMCs. Cell cycle regulation by AMPK is mediated by inhibition of the TSC2-mTOR (mammalian target of rapamycin) pathway as well as upregulation of the p53-p21 axis. Persistent activation of AMPK leads to accelerated p53-dependent cellular senescence [30], [35]. p53, a well-characterized tumor suppressor protein, is vital in multicellular organisms, where it regulates the cell cycle and induces genetic stability, apoptosis, and inhibition of angiogenesis [36]. p21 is localized to the nucleus in wild-type p53 cells undergoing G1 arrest, but not in those expressing mutant p53. Originally, p21 was thought to bind and inhibit activity of all G1 cyclin/cdk complexes. Expression of p21 is sufficient to induce growth arrest at G1 and G2 phases, even in the absence of p53. Furthermore, p21 is required for efficient G1 arrest [37]. An AMPK activator has been shown to suppress VSMC proliferation through induction of p53/p21 and subsequent downregulation of cyclin D1 [9], [22], [38], [39]. Data from the current study showed that STS significantly activates AMPK in VSMCs in a time- and dose-dependent manner. In our experiments, AMPK activation was suppressed by high glucose. STS treatment increased AMPK T172 phosphorylation in high glucose status in a dose-dependent manner (Figure 1). We further examined the effects of STS on expression of p53, a key regulator of the cell cycle downstream of AMPK, as well as p21, a downstream target of p53. STS promoted p53 and p21 expression in a time- and dose-dependent manner in VSMCs under conditions of low energy status, leading to activation of AMPK. STS has been shown to inhibit cell proliferation via p53 activation leading to p21-mediated cell cycle arrest at the G_1_ phase. p53 is instructed to favor activation of growth inhibitory genes that trigger growth arrest, and induces apoptosis in response to stress signals. However, the apoptosis-inducing effect of p53 may depend on the cell type or require further cofactors or modifications [40]. The mechanism underlying growth inhibition by STS is therefore cell cycle regulation, which is reversed by AMPK inhibition. These findings suggest that STS exerts its anti-proliferative effect, at least in part, via the AMPK-p53-p21 signaling pathway.

With our increased understanding of the cellular changes associated with vascular lesions, it is critical to determine whether inhibition of proliferation and migration of VSMCs could serve as a novel therapeutic strategy to prevent the vascular complications of diabetes. VSMC migration is increased in high glucose conditions [4], [26]. Atherosclerosis is a process of chronic inflammation, and MMPs, specifically, MMP-2 and MMP-9, are the principal enzymes in extracellular matrix degradation essential for the invasion and migration of VSMCs [18]. Moreover, expression and activity of MMP-2 are enhanced by high glucose [26], [41], which may explain the higher rate of atherosclerotic lesions in diabetic patients. In the present study, STS inhibited MMP-2 expression and activity in VSMCs induced by high glucose, but not MMP-9 activity. Furthermore, we observed a significant delay in VSMC migration into empty space upon exposure of wounded cells to STS. Our results indicate that STS inhibits high glucose-induced VSMC migration by downregulating MMP-2 synthesis, but not MMP-9. It is generally concluded that the basal MMP levels in most cell lines are low, and expression is induced during hyperglycemia via activation of transcription factors, such as NF-κB [42], [43]. AMPK activation plays a key role in the regulation of inflammation in VSMCs via suppressing NF-κB [42], [44], [45]. Moreover, activation of AMPK-mediated NF-κB suppression leads to inhibition of MMP-2 expression and activity as well as protection against cardiac remodeling [46]. Data from previous studies collectively indicate that activated AMPK inhibits VSMC migration via suppression of NF-κB and MMP. Our current experiments showed that STS inhibits VSMC migration under high glucose status via AMPK pathway. However, further studies are required to determine the cross-talk between NF-κB and AMPK.

Despite reports on the inhibitory effects of tanshinone IIA on VSMC growth and migration as well as neointimal formation in the animal model [16], [47], no studies have focused on the effects of STS under high glucose conditions mimicking diabetes. Here, we have provided preliminary evidence that STS inhibits high glucose-induced VSMC proliferation and migration partly through activation of AMPK. The growth suppression effect may be attributed to activation of AMPK-p53-p21 signaling and the inhibitory effect on migration by AMPK/NF-κB signaling axis. Moreover, the mechanism by which STS activates AMPK to suppress the NF-κB effect requires further exploration.

In summary, our results provide preliminary evidence that STS exerts an inhibitory effect on HG-induced VSMC proliferation and migration. Based on the *in vitro* findings, we examined STS-induced AMPK activation *in vivo*. Similar to the *in vitro* data, oral administration of STS led to significant activation of AMPK in mouse aorta. The increasing incidence of diabetes and subsequent vascular diseases is currently a major public health problem in industrialized countries [48]. In view of these collective findings, we propose that STS can be used extensively in diabetic patients, regardless of lipid status, for preventing atherosclerosis and restenosis after PCI. However, the current results require validation with further animal studies.

## Supporting Information

File S1 Completed ARRIVE (Animal Research: Reporting of In Vivo Experiments) Guidelines Checklist. We followed the ARRIVE guidelines for describing laboratory-based animal research and uploaded a completed ARRIVE Guidelines Checklist to be published as supporting information.
(DOC)
